# *MIR155HG* is a prognostic biomarker and associated with immune infiltration and immune checkpoint molecules expression in multiple cancers

**DOI:** 10.1002/cam4.2583

**Published:** 2019-09-30

**Authors:** Lirong Peng, Zhanfei Chen, Yiyin Chen, Xiaoqian Wang, Nanhong Tang

**Affiliations:** ^1^ Department of Hepatobiliary Surgery and Fujian Institute of Hepatobiliary Surgery Fujian Medical University Union Hospital Fuzhou Fujian China; ^2^ Key Laboratory of Ministry of Education for Gastrointestinal Cancer Research Center for Molecular Medicine Fujian Medical University Fuzhou China

**Keywords:** cancer, immune checkpoint, lncRNA *MIR155HG*, prognosis

## Abstract

In recent years, immune checkpoint inhibitor has achieved remarkable success in multiple cancer treatment. However, how to pre‐judge which patients are suitable for immune checkpoint inhibitor is a difficult problem. We use the existing public bioinformatics database to comprehensively analyze the relationship between clinical data of various cancers with immune checkpoint blocking molecules and long non‐coding RNAs (lncRNAs), and try to find the potential predictive value of lncRNA for immunotherapy with checkpoint inhibitors. In this study, we found that: (a) high expression of lncRNA *MIR155* host gene (*MIR155HG*) was closely related to better overall survival (OS) in cholangiocarcinoma (CHOL), lung adenocarcinoma (LUAD), and skin cutaneous melanoma (SKCM), and have better disease‐free survival (DFS) in CHOL. Meanwhile, the high level of *MIR155HG* was associated with poorer OS in glioblastoma multiforme (GBM), kidney renal clear cell carcinoma (KIRC), brain lower grade glioma (LGG), and uveal melanoma (UVM). (b) The expression of *MIR155HG* was significantly correlated with infiltrating levels of immune cells and immune molecules, especially with immune checkpoint molecules such as programmed cell death protein 1 (PD‐1), PD‐1 ligand 1 (PD‐L1), and cytotoxic T lymphocyte‐associated antigen 4 (CTLA4) in most kinds of cancers. (c) Detection of clinical CHOL and liver hepatocellular carcinoma tissues confirmed that there was a strong positive correlation between *MIR155HG* expression and the levels of CTLA4 and PD‐L1. *MIR155* host gene can be used as a prognostic marker in multiple cancers, and of great value in predicting the curative effect of immune checkpoint inhibitor therapy owing to it is closely related with immune cells infiltration and immune checkpoint molecules expression.

## Graphical abstract

Long non‐coding RNA *MIR155* host gene (*MIR155HG*) was closely related to overall survival in cholangiocarcinoma, lung adenocarcinoma, skin cutaneous melanoma, head and neck squamous cell carcinoma, glioblastoma multiforme, kidney renal clear cell carcinoma, brain lower grade glioma, and uveal melanoma. The expression of *MIR155HG* was significantly correlated with infiltrating levels of immune cells, molecules, and immune checkpoint molecules.

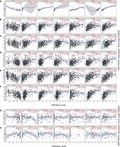

## 1 INTRODUCTION

The treatment of cancer is still a worldwide problem. The conventional therapies, including chemotherapy and radiation therapy, remain the first‐line treatment for most cancer patients. In recent years, tumor immunotherapy has shifted from adjuvant therapy to an important treatment modality with the breakthrough of checkpoint inhibitor immunotherapy.^1^

Immune checkpoint blockade treatment can give patients an unprecedented long‐lasting anti‐tumor response. By far the most widely used immunotherapy in various solid tumors and hematological malignancies is cytotoxic T lymphocyte‐associated antigen 4 (CTLA4) or programmed cell death protein 1 (PD‐1)‐PD‐1 ligand 1 (PD‐L1) inhibitor, known as immune checkpoint inhibitors (ICIs).^2^ Tumeh et al found that CD8^+^ T cell density in invasive margin, CD8^+^ T cell density in tumor, PD‐1 and PD‐L1 expression in tumor and invasive margin can be used to assess clinical response of PD‐1 inhibitor therapy.^3^ Specific changes of molecular, immunological expression, and immune infiltration in glioblastomas are associated with clinical response to anti‐PD‐1 immunotherapy.^4^ Understanding the immunophenotype and the gene expression levels of PD‐1, PD‐L1, CTLA4, and other immune checkpoint molecules are very important for cancer patients to selecting receive which kinds of immunosuppressive regimens and predicting the response of immunotherapy. Therefore, there is an urgent need to find biomarkers that can elucidate the immunophenotype in the tumor microenvironment of patients and predict immune‐related therapeutic targets.

Long non‐coding RNA (lncRNA) is a non‐coding RNA with a length of more than 200 nucleotides. It plays a complex and precise regulatory role in development and gene expression, and its mechanisms are diverse. Most of the lncRNAs have obvious space‐time expression specificity in the process of tissue differentiation and development.^5^, ^6^ Long non‐coding RNA *MIR155* host gene (*MIR155HG*), also known as B‐cell integration cluster, located in chromosome 21q21, is considered as the primary microRNA of *MIR155*.^7^, ^8^ MIR155 is involved in the expression of many immunospecific transcripts, and is up‐regulated in lymphoma. It also has oncogene activity in transgenic mouse models.^9^ *MIR155* host gene promotes glioma and GBM tumor growth,^10^, ^11^ and is associated with colorectal cancer pancreatic cancer,^12^ laryngeal squamous cell carcinoma.^13^ Multiple studies have shown that *MIR155HG* is highly expressed in diffuse large B‐cell (DLBC) lymphoma and primary mediastinal B‐cell lymphoma.^14^ In chronic lymphocytic leukemia, the transcription factor MYB activates *MIR155HG* activity, which causes the epigenetic state of *MIR155HG* to be dysregulated and causes an abnormal increase in *MIR155*.^15^ These results indicate that *MIR155HG* plays an important role in tumor progression, invasion, and metastasis. At the same time, *MIR155HG* is also thought to be involved in the human immune response. In the progression of chronic obstructive pulmonary disease, *MIR155HG* modulates M1/M2 macrophage polarization.^16^ *MIR155* host gene also modulates host innate immunity during influenza A virus infection^17^ and the transcriptional activity is activated during T‐cell activation.^18^

At present, there are few studies on the relationship between immune checkpoints and lncRNAs in tumors.^19^ In this study, we analyzed the expression of *MIR155HG* in 33 types of tumor and its relationship with prognosis and pathological staging by using the existing public bioinformatics database, and found that *MIR155HG* can be used as a prognostic biomarker in GBM, cholangiocarcinoma (CHOL), Head and Neck squamous cell carcinoma (HNSC), kidney renal clear cell carcinoma (KIRC), lower grade glioma (LGG), uveal melanoma (UVM), lung adenocarcinoma (LUAD), skin cutaneous melanoma (SKCM). We also analyzed the association of *MIR155HG* with tumor‐infiltrating immune cells and immune molecules in tumors. The results indicated that the expression of *MIR155HG* in these tumors is closely related to the immunological checkpoint molecules PD‐1, PD‐L1, CTLA4, LAG3, and TIM3. Then, the correlation between *MIR155HG* and PD‐L1 and CTLA4 was verified by clinical specimens of CHOL and liver hepatocellular carcinoma (LIHC). Therefore, we believe that *MIR155HG* can be used as a predictor for assessing the prognosis of cancer patients and the effectiveness of immunotherapy with checkpoint blockade. This finding provides an important evidence for the study of the correlation between non‐coding RNA and immunity.

## 2 MATERIALS AND METHODS

### 2.1 Tumor immune estimation resource database analysis

The Tumor immune estimation resource (TIMER) contains 10 897 samples from 32 tumor types from The Cancer Genome Atlas (TCGA) (https://cistrome.shinyapps.io/timer/), detecting the immune cell infiltration, the correlation between two genes and the differential gene expression in tumor tissues based on RNA‐Seq expression profiling data.^20^ We used the different expression pattern in TIMER to analyze the expression of *MIR155HG* in various tumor tissues and paracancer tissues. The gene pattern was used to analyze the correlation of *MIR155HG* with immune cell infiltration in various tumors, including B cells, CD8^+^ T cells, CD4^+^ T cells, macrophages, neutrophils and dendritic cells (DCs). The correlation of *MIR155HG* with NK cells, Treg cells, macrophages, DCs, mast cells, Th1, Th2, Tfh, MDSC, immunosuppressive molecules, for example CD160, CD96, CSF1R, CTLA4, HAVCR2, IDO1, IL10, IL10RB, KIR2DL1, KIR2DL3, LAG3 and immunostimulatory molecules such as BTNL2, CD27, CD28, CD40, CD40LG, CD48, CD70, CXCL12, CXCR4, ICOS, IL6, etc were analyzed by TIMER correlation modules. Spearman correlation analysis was used to assess statistical significance. Gene expression levels are expressed in log2 RSEM.

### 2.2 Gene expression profiling interactive analysis database analysis

The gene expression profiling interactive analysis (GEPIA) database integrates tens of thousands of tumor and non‐tumor samples from TCGA and GTEx gene expression data to analyze gene expression, differential gene expression, survival, correlation, and co‐expression of genes online.^21^ We analyzed the expression of *MIR155HG* in various tumors and corresponding normal tissues by GEPIA quick start tab. GEPIA survival tab perform overall survival (OS) and disease free survival (DFS) analysis of expression gene. When analyze the relation of *MIR155HG* expression and pathological staging, the group cutoff was set to median (Cutoff‐High and Cutoff‐Low are both 50%), 95% confidence interval, axis units selected months, and use major stage in the pathological stage plot of expression DIY tab. We obtained the top 80 similar expression protein‐coding genes with *MIR155HG* by similar genes module for Gene Ontology (GO), Kyoto encyclopedia of genes and genomes (KEGG) analysis.

### 2.3 GO, KEGG analysis by Cytoscape

Cytoscape is an open data resource that integrates high‐throughput expression data and other types of data into biomolecular interaction networks.^22^ Functional enrichment and path enrichment analysis were performed with ClueGO^23^ and CluePedia^24^ in Cytoscape. The 80 protein‐coding genes most consistent to *MIR155HG* expression were subjected to GO analysis include molecular function, biological process, cellular component enrichment (*P* ≤ .001), and KEGG Pathway (*P* ≤ .05) network.

### 2.4 Clinical samples

The frozen resected specimens from 10 patients with CHOL and 31 patients with LIHC were obtained from Fujian Medical University Union Hospital from October 1998 to July 2018 for CHOL and August 2009 to April 2019 for LIHC. The collection of samples for research use was approved by the Institution Review Board of the College of Life Sciences, Fujian Medical University in accordance with guide‐lines for the protection of human subjects.

### 2.5 RNA extraction and real‐time RT‐PCR analysis

Total RNAs were extracted from frozen tissue of patients by trizol reagent (Invitrogen) and strictly followed the operation instructions. The reverse transcription reaction was carried out using a cDNA synthesis kit (Roche). Quantitative real‐time PCR was performed according to manufacturer's instructions, with the ABI 7500 Real‐Time System (Life Technologies) and the EVA Green fluorescence (ABM, Vancouver, Canada). The reactions were performed as described previously.^25^ The relevant primer sequences are given in Table S1, and glyceraldehyde‐3‐phosphate dehydrogenase transcripts were used as a reference. Data were analyzed using the comparative Ct method by use of the $2^{-\Delta C_{t}}$ method.

### 2.6 Statistical analysis

Spearman's rho correlation coefficient was used to evaluate a possible correlation between the two genes. The statistical analyses were performed with SPSS software (IBM SPSS Statistics 20). The figures were performed with GraphPad Prism 5.01.

## 3 RESULTS

### 3.1 Expression levels of *MIR155HG* in different types of tumors

**Figure 1:**
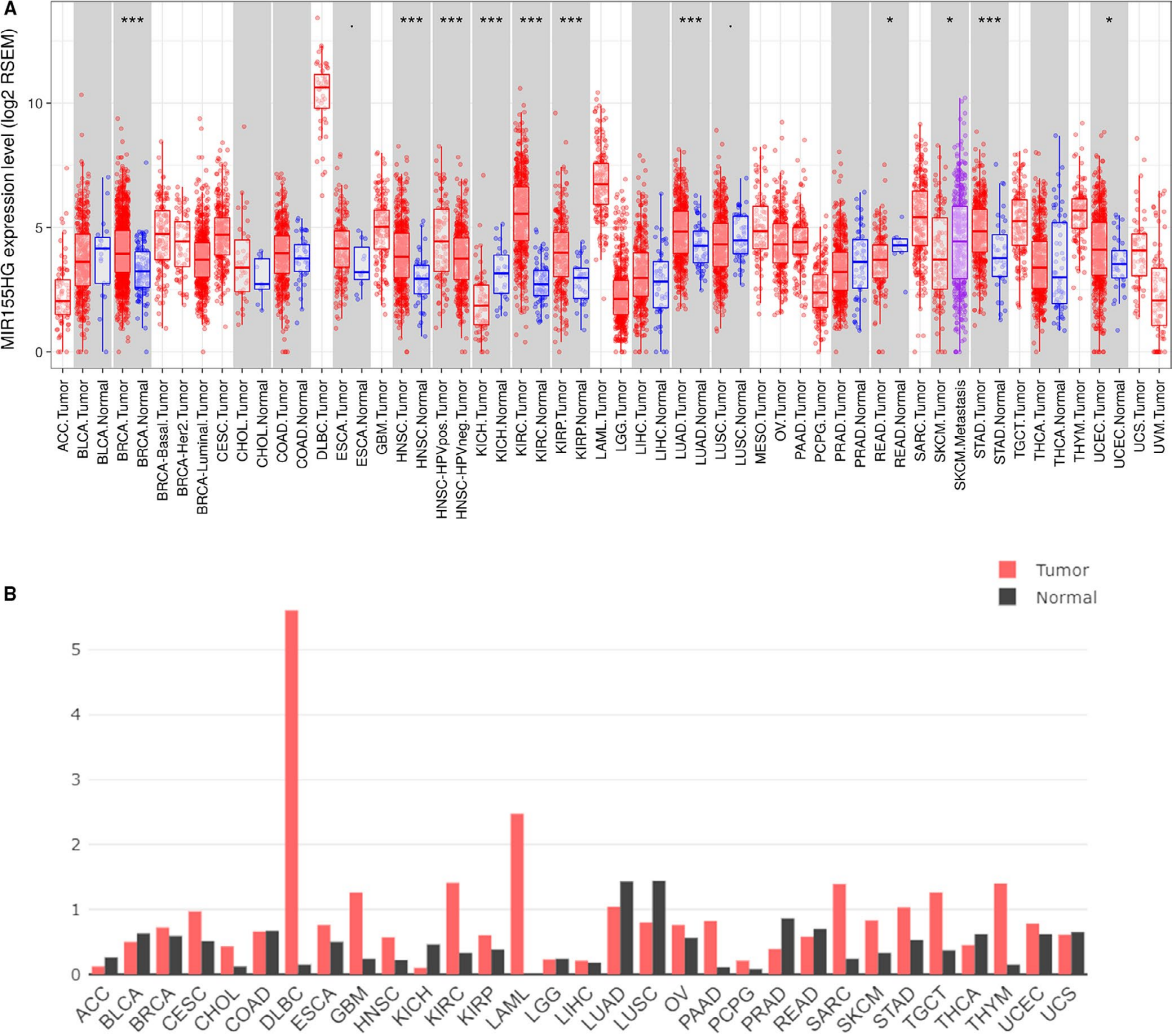
Expression levels of *MIR155* host gene (*MIR155HG*) in different kinds of cancers. A, The *MIR155HG* expression in different tumor types with or without paracancer determined by TIMER (*P*‐value significant codes: 0≤ *** <.001 ≤ ** <.01 ≤ * <.05 ≤. <.1). B, Increased or decreased *MIR155HG* in different cancers compared with paired normal tissues by gene expression profiling interactive analysis

The TIMER online database was used to analyze the differential expression of *MIR155HG* in 17 types of tumors and adjacent tissues in TCGA, and the differential expression was evaluated by Wilcoxon test. The results showed that, compared to the paracancerous control, *MIR155HG* was highly expressed in breast invasive carcinoma, HNSC, KIRC, kidney renal papillary cell carcinoma, LUAD, stomach adenocarcinoma and uterine corpus endometrial carcinoma, lower expression in kidney chromophobe (KICH), rectum adenocarcinoma (*P* < .05) (Figure 1A). The GEPIA database analyzes the expression of *MIR155HG* in various tumors and matched normal tissues (match TCGA normal and GTEx data), and the results showed that *MIR155HG* was higher than the matched normal tissue in the lymphoid neoplasm DLBC lymphoma, GBM, KIRC, acute myeloid leukemia, thymoma (cutoff criteria: |log_2_fold change| > 1.0 and *P* < .05) (Figure 1B).

### 3.2 *MIR155HG* was associated with the prognosis in a variety of tumors

**Figure 2:**
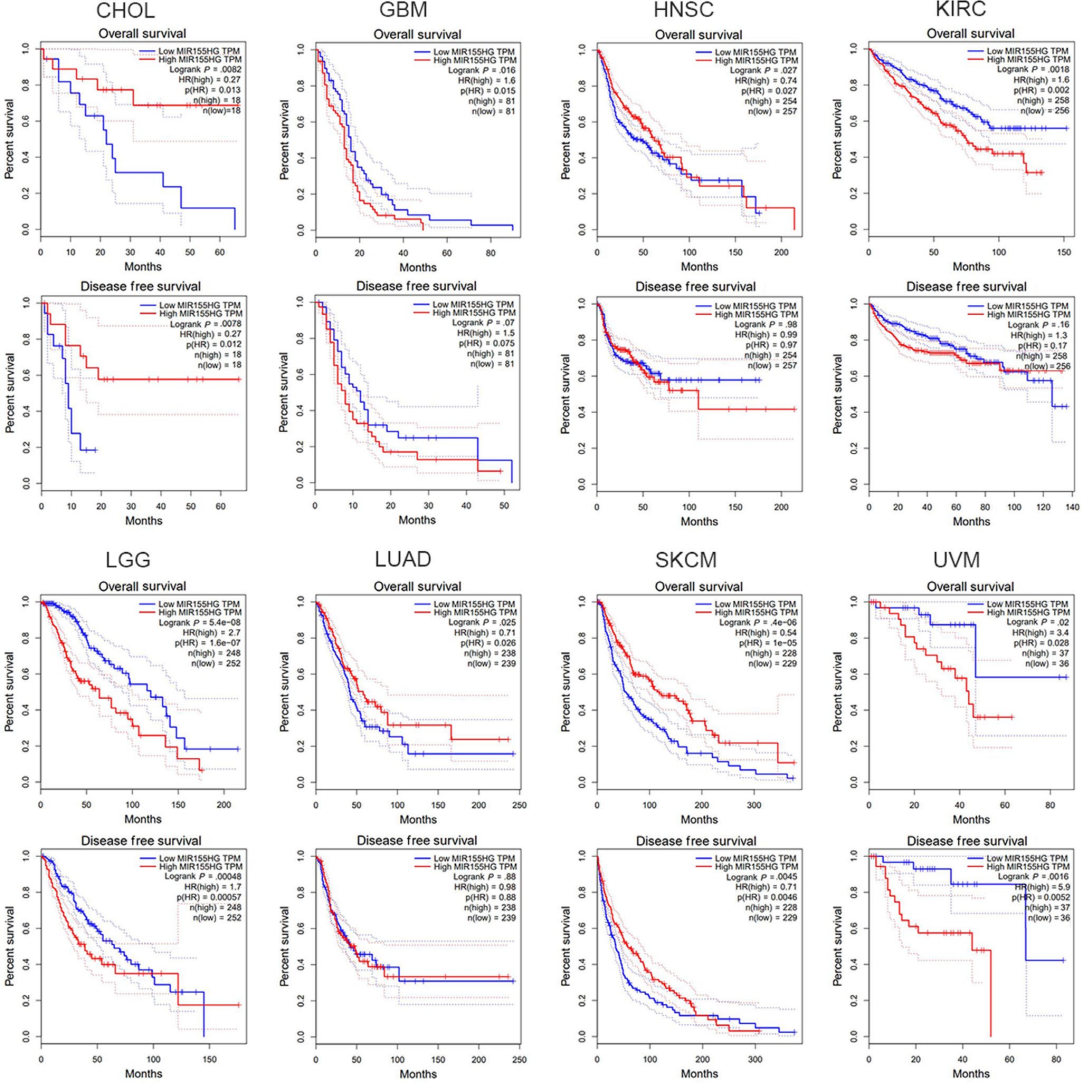
Prognostic value of *MIR155* host gene (*MIR155HG*) in cancer patients. Kaplan‐Meier survival curves comparing the overall survival and disease free survival of the high and low expression of *MIR155HG* in cholangiocarcinoma (CHOL), GBM, head and neck squamous cell carcinoma (HNSC), kidney renal clear cell carcinoma (KIRC), lower grade glioma (LGG), lung adenocarcinoma (LUAD), skin cutaneous melanoma (SKCM), and uveal melanoma (UVM) (gene expression profiling interactive analysis database)

We used GEPIA to analyze the relation of *MIR155HG* and clinical features in 33 kinds of tumors and found that *MIR155HG* was correlated with OS, DFS, and staging in multiple tumors. The *MIR155HG* higher expression has better OS and DFS in CHOL and prophase SKCM. In LUAD and early stage of HNSC, patients with high levels of *MIR155HG* have better OS than patients with low expression of *MIR155HG*. While high levels of *MIR155HG* was associated with poor OS in GBM, KIRC, LGG, and UVM, and poor DFS in LGG early stage and UVM *MIR155HG* was closely related to tumor stage in KICH, KIRC, LUAD, SKCM, Thyroid carcinoma (THCA) (Figure 2; Table S2).

### 3.3 Gene enrichment analysis mRNAs high correlated with *MIR155HG*

**Figure 3:**
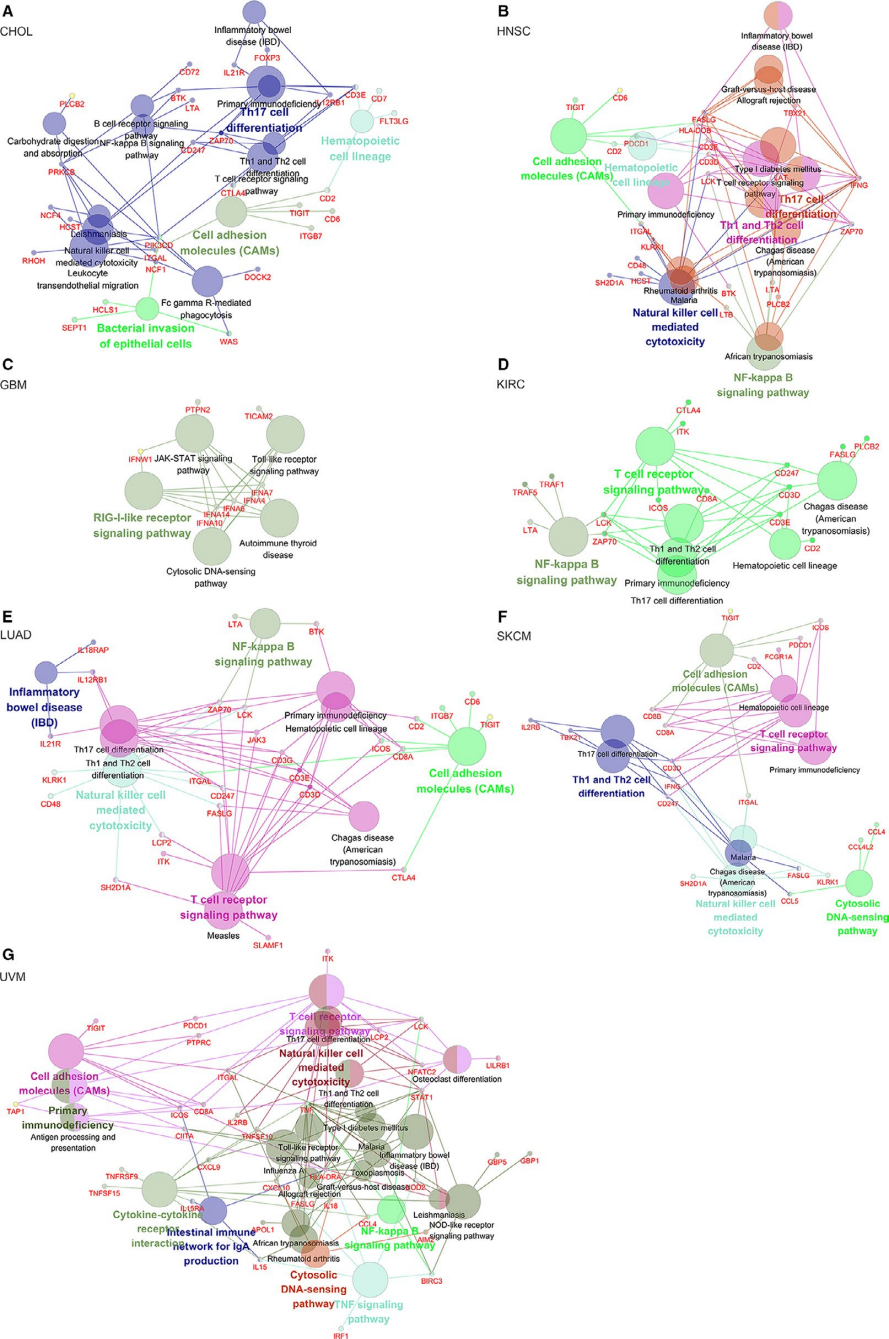
Kyoto encyclopedia of genes and genomes pathway analysis of the *MIR155* host gene (*MIR155HG*) co‐expression gene. The circle indicate the terms that are significantly enriched in various tumors, the larger indicates higher degree of enrichment. Different colors represent the clustering of different terms. A, Cholangiocarcinoma (CHOL), (B) head and neck squamous cell carcinoma (HNSC), (C) GBM, (D) kidney renal clear cell carcinoma (KIRC), (E) lung adenocarcinoma (LUAD), (F) skin cutaneous melanoma (SKCM), (G) uveal melanoma (UVM) (show only pathways with pV ≤ 0.05)

According to the correlation between *MIR155HG* and OS or DFS in eight types of tumors (CHOL, HNSC, GBM, KIRC, LGG, LUAD, SKCM, UVM), the gene pathway enrichment and functional enrichment were further analyzed. The top 80 mRNAs co‐expressed with *MIR155HG* in various tumors were obtained by GEPIA's similar genes model. GO and KEGG analysis was performed using the ClueGO and CluePedia in Cytoscape software. GO analysis showed that a large number of genes were significantly enriched in the immune‐related process in CHOL, HNSC, GBM, KIRC, LUAD, SKCM, and UVM, but there was no immune‐related term in LGG (*P* ≤ .001) (Figure S1). Kyoto encyclopedia of genes and genomes results show that there were abundant genes enriched in immune‐related pathways, such as T‐cell receptor signaling, Th1, Th2, Th17 cell differentiation, NK cell‐mediated cytotoxicity, and other pathways in CHOL, HNSC, LUAD, SKCM, and UVM. There was also a certain amount of gene enrichment in immune function‐related pathways in KIRC, but less in GBM, and no meaningful pathways in LGG (*P* ≤ .05) (Figure 3).

### 3.4 Correlation between *MIR155HG* and immune cells and immune molecules

**Figure 4:**
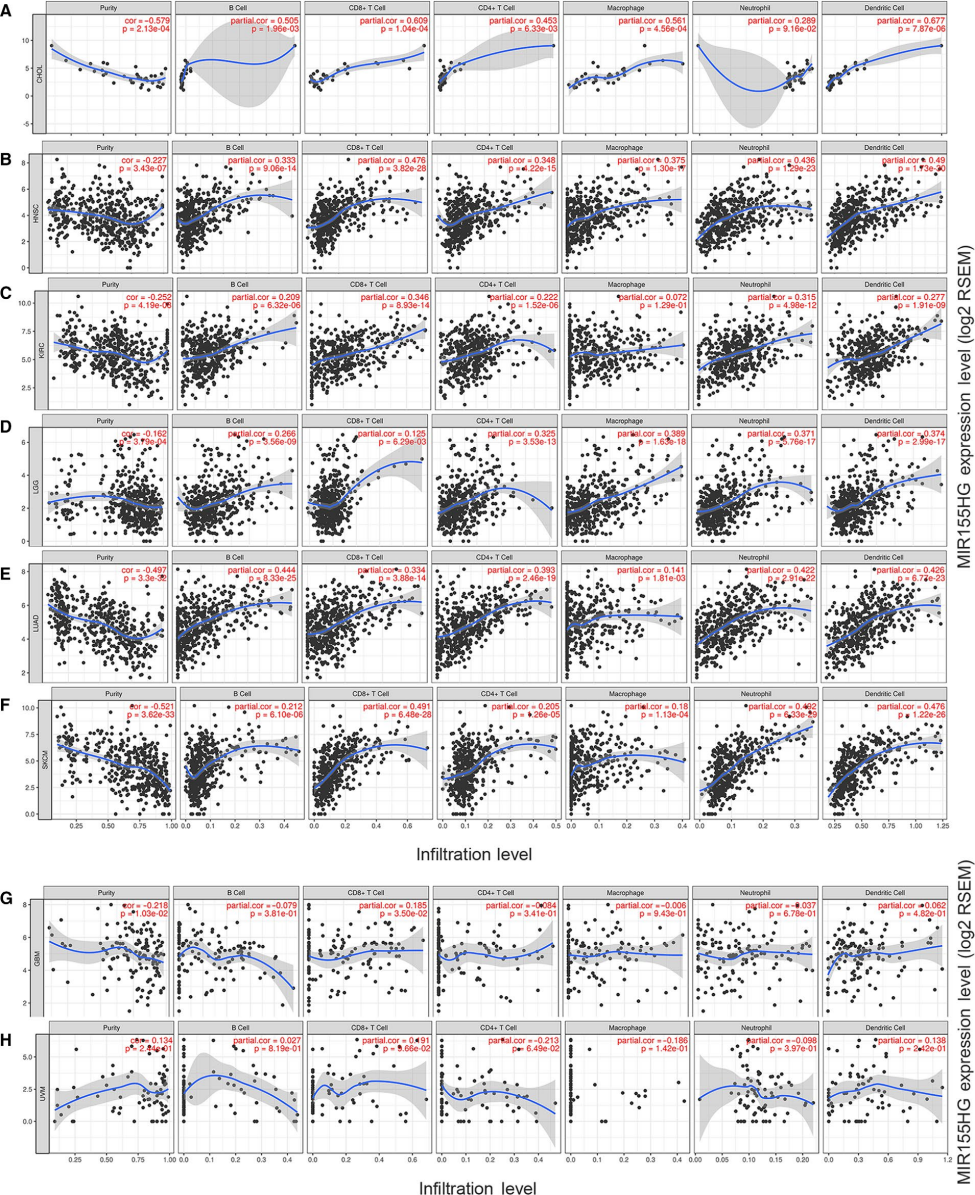
Correlation of *MIR155* host gene (*MIR155HG*) expression with immune infiltration level by TIMER in eight kinds of cancer. A‐F, *MIR155HG* expression is negatively correlated with tumor purity and has significant positive relates with infiltrating levels of B cells, CD8^+^ T cells, CD4^+^ T cells, and dendritic cells (DCs) in cholangiocarcinoma (CHOL), head and neck squamous cell carcinoma (HNSC), kidney renal clear cell carcinoma (KIRC), lower grade glioma (LGG), lung adenocarcinoma (LUAD), skin cutaneous melanoma (SKCM), but has no significant correlations with infiltrating levels of neutrophils in CHOL, macrophages in KIRC. G, *MIR155HG* is showed a very weak negatively correlated with tumor purity and positive relates with infiltrating levels of CD8^+^ T cells in GBM (.01 < *P* < .05). H, *MIR155HG* expression is no significant correlation with tumor purity and infiltrating level of B cells, CD8^+^ T cells, CD4^+^ T cells, macrophages, neutrophils, and DCs in uveal melanoma (UVM)

To investigate the correlation between *MIR155HG* and tumor immune cells, we used the TIMER to analyze the correlation of *MIR155HG* with tumor purity, lymphocytes, macrophages, neutrophils, DCs, NK cells, Treg cells, mast cells, Th1, Th2, Th17, Tfh cells, MDSC in the above eight tumors. The results showed that the expression of *MIR155HG* in CHOL, HNSC, KIRC, LGG, LUAD, and SKCM was significantly correlated with tumor purity and the infiltration level of immune cells such as B lymphocytes, CD8^+^ T cells, CD4^+^ T cells and DCs. The correlation with immune cells was relatively weaker in GBM and UVM (Figure 4). *MIR155* host gene was highly correlated with NK cells, Treg cells, macrophages, Th1, Tfh cells and M‐MDSC in CHOL, HNSC, KIRC, LUAD, and SKCM, and have a certain correlation with MDSC in GBM *MIR155HG* was related to the infiltration of macrophages, Th1 and DMSC in a certain degree in LGG, and has moderate correlated with Treg cells, Th1 and Tfh cells in UVM (Table S3).

To further understand the ability of *MIR155HG* to assess immune status in vivo, we further analyzed the correlation of *MIR155HG* with PDCD1, CD274, CTLA4, IL10, IDO1, TGFB1 and other immunosuppressive molecules and with immunostimulatory molecules such as CD80, CD86, CD27, CD28, CXCR4 CXCL12, ICOS *MIR155HG* was found to have a moderate to strong positive correlation with immunosuppressive molecules CD96, CTLA4, LAG3, PDCD1, TIGIT and with immunostimulatory factors CD27, CD28, CD40LG, CD48, CXCR4, ICOS, LTA, TMIGD2, CD80, and CD86 in CHOL, HNSC, KIRC LUAD, and SKCM (Table S4).

### 3.5 Correlation between *MIR155HG* and immunological checkpoint blocking molecules

**Figure 5:**
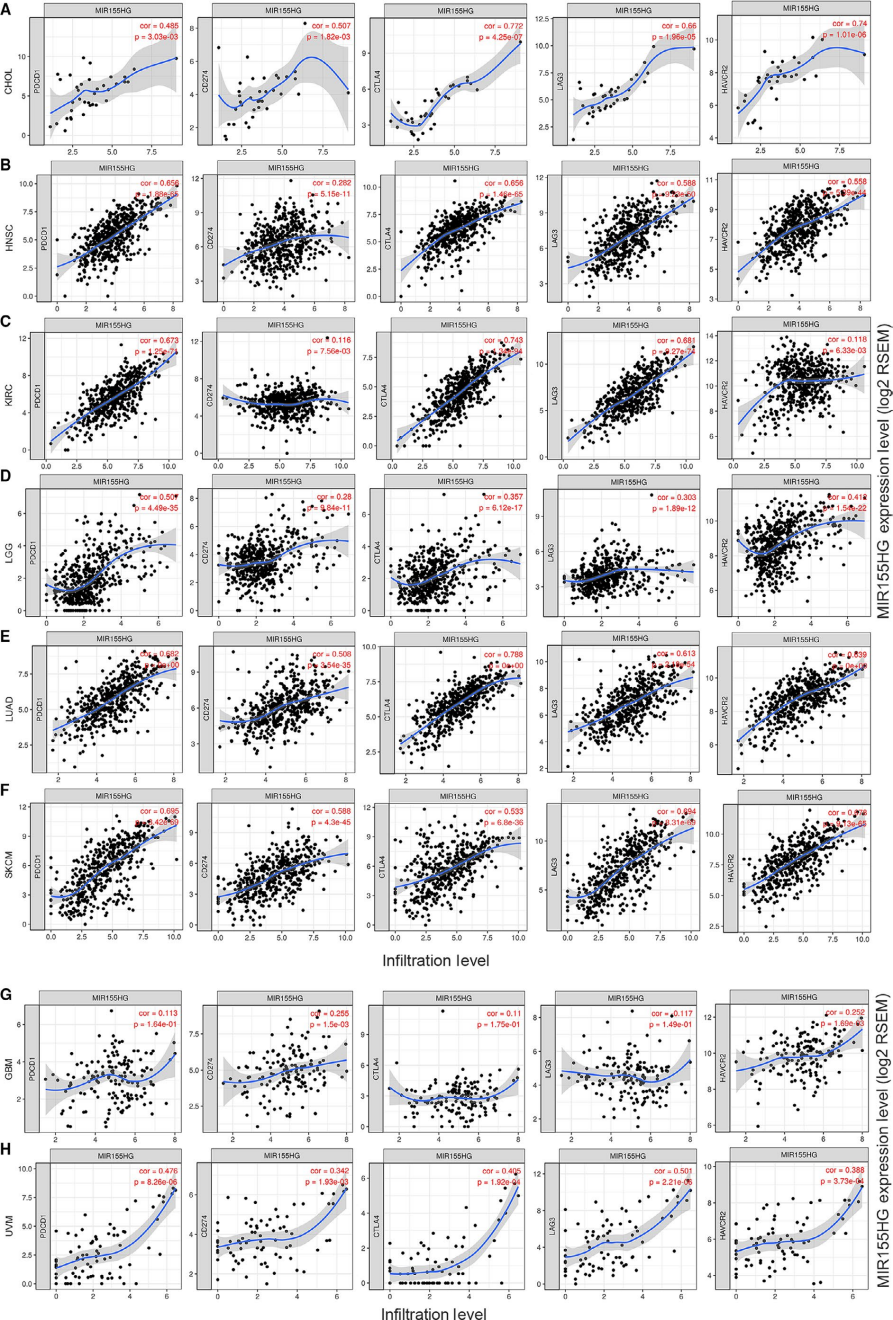
Correlation of *MIR155HG* expression with immune checkpoints molecules.(A‐F, H) *MIR155* host gene (*MIR155HG*) expression are positively correlated with the mRNA levels of programmed cell death protein 1 (PD‐1) (CD274), PD‐1 ligand 1 (PD‐L1) (CD274), cytotoxic T lymphocyte‐associated antigen 4 (CTLA4), LAG3 and TIM3 (HAVCR2) in cholangiocarcinoma (CHOL), head and neck squamous cell carcinoma (HNSC), kidney renal clear cell carcinoma (KIRC), lower grade glioma (LGG), lung adenocarcinoma (LUAD), skin cutaneous melanoma (SKCM) and uveal melanoma (UVM). G, *MIR155HG* expression is weak correlated with PD‐L1 and TIM3 but uncorrelated with PD‐1, CTLA4, and LAG3 in GBM

To evaluate the efficacy of *MIR155HG* in predicting cancer patient response to checkpoint inhibitor, we used the TIMER database to analyze the relevance of *MIR155HG* and the currently available blocking molecules with superior therapeutic effects PD‐1, PD‐L1, CTLA4, LAG3, TIM3. A significant strong positive correlation (cor > 0.5) was found between *MIR155HG* with PD‐1 (PDCD1), PD‐L1 (CD274), CTLA4, LAG3, and TIM3 (HAVCR2) molecules in LUAD and SKCM, and with a median or higher correlation in CHOL and UVM patients (cor > 0.3), a significant correlation in HNSC, LGG (*P* < .0001). There was a strong positive correlation with PD‐1, CTLA4, and LAG3 in KIRC, and a certain correlation with PD‐L1 and TIM3 in GBM (Figure 5).

### 3.6 Relationship between *MIR155HG* and immunity in other type of tumors

**Figure 6:**
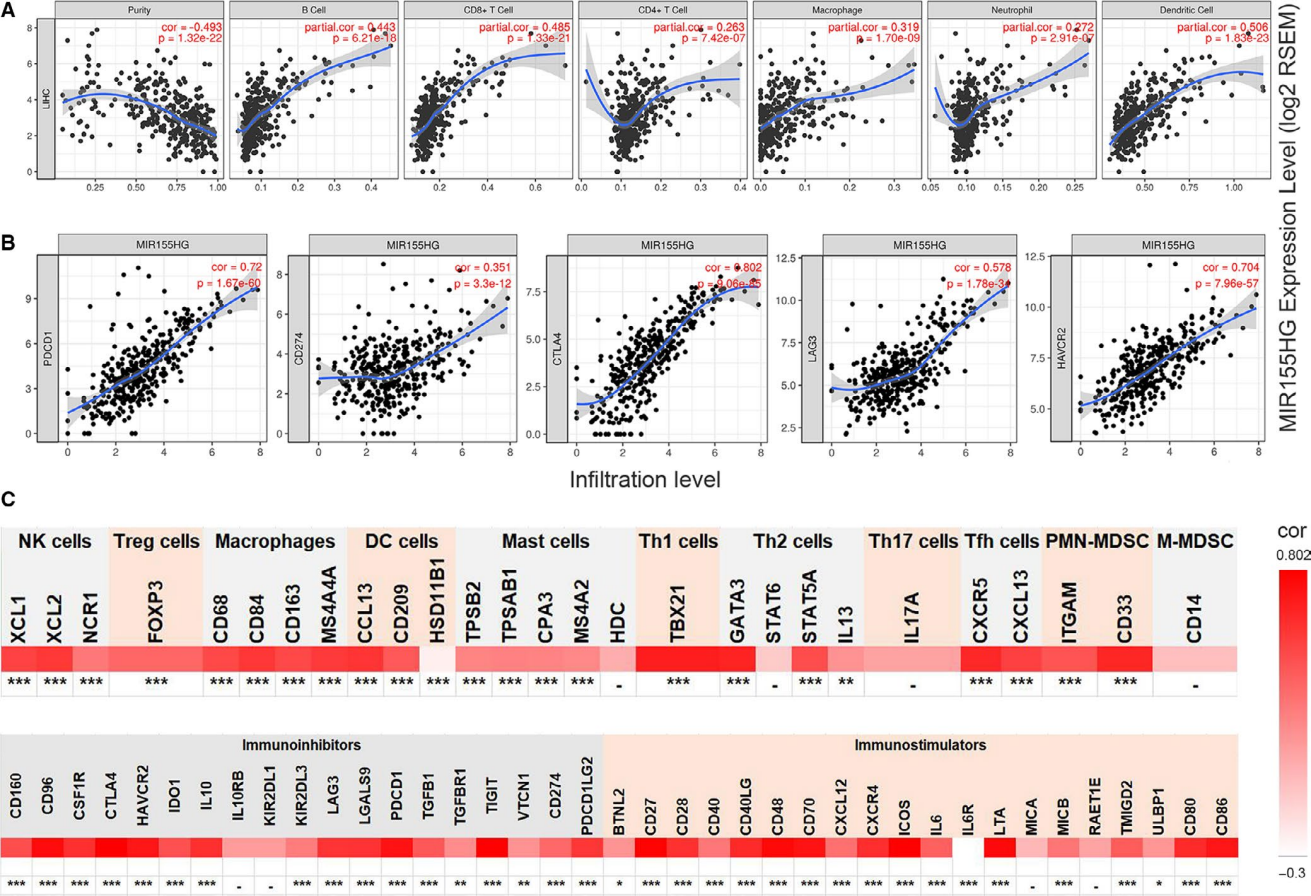
Relationship between *MIR155* host gene (*MIR155HG*) and immune cells, molecules and immune checkpoint molecules in hepatocellular carcinoma. A, *MIR155HG* is inversely correlated with tumor purity and is significantly positively correlated with B cells, CD8^+^ cells, CD4^+^ cells, macrophages, neutrophils, and dendritic cells (DCs). B, *MIR155HG* was significantly positively correlated with programmed cell death protein 1 (PD‐1), CD274, cytotoxic T lymphocyte‐associated antigen 4 (CTLA4), LAG3, and TIM3. C, Correlation between *MIR155HG* and immune molecular gene expression (0 ≤ *** < .0001 ≤ ** < .001 ≤ * < .01≤ ‐)

The relationship between *MIR155HG* and immunity was relatively close in the eight types of prognostic‐related tumor mentioned above, and what is the relationship with other types of tumors that unrelated to prognosis? Therefore, we conducted further study in LIHC. The results showed that *MIR155HG* was significantly negative associated with tumor purity, positive correlated with B cells, CD8^+^ T cells, CD4^+^ T cells, macrophages, neutrophils and DCs. In LIHC, *MIR155HG* has a high correlation with immunological checkpoint molecules PD‐1, CTLA4, LAG3, and TIM3 (cor > 0.5), and also has a significant correlation with PD‐L1. *MIR155* host gene with NK, Treg, Th1, Tfh cells, and PMN‐MDSC and most of the immune molecules have a significant correlation (Figure 6). We also analyzed the relationship between *MIR155HG* and immune in other tumors, and found that *MIR155HG* is closely related to immune cells and molecules in most kind of tumors (Figures S2 and S3).

### 3.7 Verify the correlation between *MIR155HG* and PD‐L1, CTLA4 by clinical specimens

**Figure 7:**
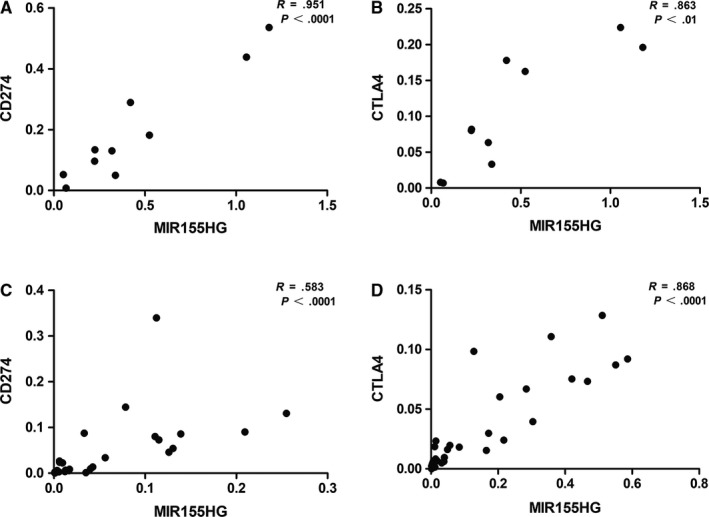
Correlation between *MIR155* host gene (*MIR155HG*) and CD274 (programmed cell death protein 1 [PD‐1]), cytotoxic T lymphocyte‐associated antigen 4 (CTLA4) gene expression in clinical specimens. A and B, The figure shows *MIR155HG*, CD274, and CTLA4 absolute mRNA expression values measured in 10 cases of cholangiocarcinoma. C and D, The correlation was measured in 31 cases of liver hepatocellular carcinoma

Since the great value of *MIR155HG* predicting the immune checkpoint molecular expression level in tumor, we selected the prognostic‐related tumor type CHOL and the prognostic‐unrelated tumor type LIHC to detect the correlation. The relationship of *MIR155HG* and the immunological checkpoint molecules PD‐L1 and CTLA4 were verified by qRT‐PCR. The results showed that *MIR155HG* showed a striking positive correlation with both PD‐L1 and CTLA4 in CHOL and LIHC (Figure 7).

## 4 DISCUSSION

In this report, we analyzed the expression of *MIR155HG* in various cancers and paracancer or normal tissues, and analyzed the relationship between *MIR155HG* expression and OS, DFS and staging, consistent with Wu's finding that *MIR155HG* was associated with poor tumor prognosis in glioma.^10^ As reported, the expression of *MIR155HG* was significantly higher in cancer than paracancer in KIRC,^26^ and *MIR155HG* was associated with poor OS.^27^ GO and KEGG analysis in these types of tumors showed that mRNAs co‐expressed with *MIR155HG* were mostly enriched in immune‐related functions and immune‐related pathways, which indicated that *MIR155HG* may be related to immunity.

The immune cells play important roles in tumor immune network and tumor development,^28^, ^29^, ^30^ previous studies have focused on describe the relationship of new antigens and survival by genomic data from TCGA,^31^ and committed to describing the immune infiltration cellular composition in tumors. For example, RNA expression data were used to analyze the infiltration of B cells, T cells, and macrophages, as well as CD4^+^ T cells, CD8^+^ T cells, neutrophils, macrophages, and DCs, multiple immune cell subsets, and multiple immune molecules.^32^, ^33^, ^34^ In fact, it is particularly important to comprehensively know the immune infiltration status on cancer patients in order to choose the correct immunotherapy strategy individually. In this study, we examined the association of *MIR155HG* with immune cells attempting to reveal the immune status in cancer patients by understanding the expression of *MIR155HG*. The results showed that *MIR155HG* was significantly negatively correlated with tumor purity and significantly positively correlated with B cells, CD8^+^ T cells, CD4^+^ T cells, and DCs in most kinds of cancers. Moreover, to fully demonstrate the relationship between *MIR155HG* and immunity, we also analyzed the association of *MIR155HG* with immunosuppressive molecules and immunostimulatory molecules in the above described cancer types. It was found that there was a high correlation with most immunosuppressive and immunostimulatory molecules in HNSC, KIRC, LGG, LUAD, and SKCM. In recent years, with the breakthroughs in the research of immunological checkpoint, the targeted inhibitors have been used in the field of cancer immunotherapy, showing unique effects. The humanized anti‐CTLA4 antibody ipilimumab was approved by the US Food and Drug Administration for clinical treatment of metastatic melanoma in 2011.^35^ At the same time, PD‐1‐PD‐L1‐axis antibody has been approved for second‐line or first‐line therapy for the treatment of melanoma, lymphoma, lung cancer, renal cell carcinoma, head and neck squamous cell carcinoma, bladder cancer, liver cancer, and gastroesophageal cancer.^36^ However, most of the patients who receive immunological checkpoint block treatment did not benefit from it.^35^ Herbst et al conducted a large sample of clinical randomized controlled trials, pembrolizumab achieves extended OS and has a favorable benefit‐to‐risk profile in patients with PD‐L1 positive advanced non‐small‐cell lung cancer,^37^ and a phase II basket study (KEYNOTE‐158) investigated the antitumor activity and safety of pembrolizumab in multiple cancer types, concluded that in previously treated advanced cervical cancer: PD‐L1–positive tumors have a stronger response after pembrolizumab treatment.^38^ Although the expression of PD‐L1 at the tumor site can predict the patient's reactivity to PD‐1 blockers, PD‐L1 expression status alone is insufficient in determining which patients should be accepted PD‐1 or PD‐L1 blockade therapy.^39^ The dynamic nature of the immune microenvironment, other checkpoint molecules, tumor infiltrating cells, immune biomarkers, mutational load are critical in immunotherapy response.^40^ Therefore, comprehensive and precision pre‐biomarkers are important for clinically personalized immunotherapy. We found that *MIR155HG* was significantly associated with immunological checkpoint blocking molecules PD‐1, PD‐L1, CTLA4, LAG3, and TIM3 in many tumors, and some of those types of tumor have better reactivity against immunological checkpoint blockade. Therefore, we speculated *MIR155HG* can be used to predict the effectiveness of immune blockade therapy among the types of tumors patients that were effective in immunotherapy monitoring block therapy.

It has been reported that the immune status of the tumor microenvironment and the molecular expression of the immune checkpoint can predict the immunotherapy reactivity of patients.^3^ However, there are many kinds of immune molecules in the tumor microenvironment, and it is of great clinical value to find a molecule that can comprehensively reflect the immune scene in the tumor. At present, there are few studies on predicting the overall immune status of the body by detecting a single molecule. Pan et al studied the prognostic value of LAYN in gastric cancer and colon cancer and its correlation with immune infiltration. They found that the expression of LAYN was associated with increased levels of immune permeation of CD8^+^ T cells, CD4^+^ T cells, macrophages, neutrophils, and DCs in colon and gastric cancer.^41^ Compared with the results of Pan et al, *MIR155HG* has a wider range of tumor applicability and was more closely related to immune cells and immune molecules. The previous research reported that *MIR155HG* participate the regulator of innate immunity^17^ and macrophage polarization,^16^ and associated with acute rejection, T‐cell‐mediated acute rejection and graft loss.^42^ *MIR‐155* regulates the expression of many immune‐specific transcripts, such as regulate polarization of macrophages, DCs maturation, T‐cell differentiation, controls B cell proliferation and antibody production.^43^, ^44^ Consider that *MIR155* is derived from *MIR155HG* and that *MIR155HG* play a critical role by interaction with *MIR155*,^9^, ^10^ we speculated that *MIR155HG* may affect the immune process through its interaction with *MIR155* or other mechanisms.

To preliminarily verify the relationship between *MIR155HG* and immune infiltration in an individual patient, we verified the correlation between *MIR155HG* and immune checkpoint molecules PD‐L1 and CTLA4 by realtime quantitative reverse transcription polymerase chain reaction (qRT‐PCR) in frozen tissue specimens of patients with CHOL and LIHC. There was a significant positive correlation between the expression of *MIR155HG* and PD‐L1, CTLA4 in clinical specimens. However, due to the small number of clinical specimen and the fewer types of tumors types, it is necessary to expand the research. The mechanism of interaction between *MIR155HG* and immune molecules needs further investigation. In conclusion, the above results indicate that *MIR155HG* expression might help to predict the prognosis and understanding immune status in cancer.

In this study, we found that *MIR155HG* can be used as a biomarker of prognosis in CHOL, GBM, HNSC, KIRC, LGG, LUAD, SKCM, and UVM through bioinformatics analysis. And the expression level of *MIR155HG* has a certain correlation with immune molecules in various types of tumors, especially in HNSC, LUAD, KIRC, SKCM, and LIHC, which are suitable for predicting the curative effect of immune checkpoint blockade therapy. This study provides an important basis for the clinical to use *MIR155HG* as an evaluation of patients' immune status and the choice of individualized immunotherapy regimen.

## CONFLICT OF INTERESTS

None declared.

## AUTHOR CONTRIBUTIONS

LP and NT conceived and designed the study. LP and ZC performed the bioinformatics analyses and experiments. LP and ZC analyzed the data. YC and XW contributed medical materials. LP prepared the original draft. NT made revisions and supervised the project. All authors support the publication of the manuscript.

## Data Availability

This article permits use, distribution, and reproduction in any medium, provided the original work is properly cited.
