# Supplementary material for: MIR155HG is a prognostic biomarker and associated with immune infiltration and immune checkpoint molecules expression in multiple cancers
Source: Cancer Med. 2019 Sep 30;8(17):7161–73. doi: 10.1002/cam4.2583 (PMC6885872; doi:10.1002/cam4.2583)
Supplement: Supplementary file 1 [file CAM4-8-7161-s001.pdf]

Supplementary Materials:

TABLE S1 List of primer used in Real-time RT-PCR of the present study

Gene names	Sequences (5’→3’)
MIR155HG-F	GTCCCAAATCTAGGTTCAAGTTCA
MIR155HG-R	TCTCTATCTCATCTAAGCCTCACAA
CD274-1F	GCCGAAGTCATCTGGACAAG
CD274-1R	AGTTGTTGTGTTGATTCTCAGTGT
CTLA4-F	CATGATGGGGAATGAGTTGACC
CTLA4-R	TCAGTCCTTGGATAGTGAGGTTC
GAPDH-F	AGGGCTGCTTTTAACTCTGGT
GAPDH-R	TCTCGCTCCTGGAAGATGGTG

TABLE S2 Correlation of *MIR155HG* mRNA expression and clinical prognosis, pathological stage in 33 types of cancer

Types	Overall survival					Disease free survival					Pathological stage	
of			Logrank					Logrank p			F	
cancer	n(high)	n(low)	p	HR(high)	p(HR)	n(high)	n(low)		HR(high)	p(HR)	value	Pr(>F)
ACC	38	29	0.29	1.6	0.29	38	29	0.63	1.2	0.64	1.05	0.377
BLCA	198	199	0.98	0.99	0.97	198	199	0.6	0.91	0.6	2.19	0.114
BRCA	533	533	0.33	0.85	0.33	533	533	0.8	1	0.8	1.98	0.0953
CESC	144	146	0.13	0.7	0.13	144	146	0.13	0.64	0.13	0.975	0.405
CHOL	18	18	**0.0082**	0.27	**0.013**	18	18	**0.0078**	0.27	**0.012**	0.503	0.609
COAD	134	131	0.4	1.2	0.4	134	131	0.68	1.1	0.68	0.27	0.847
DLBC	23	23	0.36	1.9	0.37	23	23	0.1	2.9	0.12	0.523	0.669
ESCA	91	91	0.38	1.2	0.38	91	91	0.62	1.1	0.61	1.64	0.181
GBM	81	81	**0.016**	1.6	**0.015**	81	81	0.07	1.5	0.075	-	-
HNSC	254	257	**0.027**	0.74	**0.027**	254	257	0.98	0.99	0.97	0.0458	0.987
KICH	26	32	0.6	0.69	0.6	26	32	0.71	1.3	0.71	3.95	**0.0122**
KIRC	258	256	**0.0018**	1.6	**0.002**	258	256	0.16	1.3	0.17	9.21	**6.10E-06**
KIRP	141	140	0.31	1.4	0.31	141	140	0.5	1.2	0.5	0.415	0.742
LAML	53	53	0.15	1.5	0.15	53	53	1	1	NaN	-	-
LGG	248	252	**5.40E-08**	2.7	**1.60E-07**	248	252	**0.00048**	1.7	**0.00057**	-	-
LIHC	178	174	0.87	1	0.86	178	174	0.54	1.1	0.54	0.485	0.693
LUAD	238	239	**0.025**	0.71	**0.026**	238	239	0.88	0.98	0.88	3.1	**0.0267**
LUSC	241	237	0.93	0.99	0.94	241	237	0.69	0.93	0.69	0.816	0.485
MESO	41	41	0.12	1.5	0.11	41	41	0.55	1.2	0.55	-	-
OV	209	208	0.11	0.82	0.11	209	208	0.78	0.97	0.78	0.667	0.514
PAAD	89	89	0.55	0.88	0.56	89	89	0.65	1.1	0.66	1.54	0.206
PCPG	91	91	0.73	0.75	0.73	91	91	0.55	0.74	0.55	-	-
PRAD	240	244	0.81	0.86	0.82	240	244	0.2	1.3	0.2	-	-
READ	46	46	0.44	0.69	0.45	46	46	0.4	1.5	0.41	0.906	0.442
SARC	131	131	0.4	1.2	0.4	131	131	0.44	1.1	0.44	-	-
SKCM	228	229	**7.40E-06**	0.54	**1.00E-05**	228	229	**0.0045**	0.71	**0.0046**	3.18	**0.0137**
STAD	191	192	0.34	0.86	0.34	191	192	0.9	0.98	0.91	2.06	0.105
TGCT	68	68	0.079	6.10E+08	1	68	68	0.94	1	0.95	2.68	0.0722
THCA	255	248	0.67	1.2	0.67	255	248	0.91	1	0.91	6.23	**0.00037**
THYM	59	59	0.18	2.8	0.2	59	59	0.15	1.9	0.16	-	-
UCEC	86	85	0.38	0.73	0.38	86	85	0.36	0.73	0.36	1.44	0.232
UCS	27	27	0.14	0.58	0.14	27	27	0.78	0.91	0.79	0.568	0.639
UVM	37	36	**0.02**	3.4	**0.028**	37	36	**0.0016**	5.9	**0.0052**	-	-

ACC, adrenocortical carcinoma; BLCA, bladder urothelial carcinoma; BRCA, breast invasive carcinoma; CESC, cervical squamous cell carcinoma and endocervical adenocarcinoma; CHOL, cholangio carcinoma; COAD, colon adenocarcinoma; DLBC lymphoid neoplasm diffuse large B-cell lymphoma; ESCA, esophageal carcinoma; GBM glioblastoma multiforme; HNSC, head and neck squamous cell carcinoma; KICH, kidney chromophobe; KIRC, kidney renal clear cell carcinoma; KIRP, kidney renal papillary cell carcinoma; LAML, acute myeloid leukemia; LGG, lower grade glioma; LIHC, liver hepatocellular carcinoma; LUAD, lung adenocarcinoma; LUSC, lung squamous cell carcinoma; MESO, mesothelioma; OV, ovarian serous cystadenocarcinoma; PAAD pancreatic adenocarcinoma; PCPG, pheochromocytoma and paraganglioma; PRAD prostate adenocarcinoma; READ, rectum adenocarcinoma; SARC, sarcoma; SKCM, skin cutaneous melanoma; STAD, stomach adenocarcinoma; TGCT , testicular germ cell tumors; THCA, thyroid carcinoma; THYM, thymoma; UCEC, uterine corpus endometrial carcinoma; UCS, uterine carcinosarcoma; UVM, uveal melanoma.

TABLE S3 Correlation analysis between *MIR155HG* and relate markers of immune cells

	Gene	CHOL		GBM		HNSC		KIRC		LGG		LUAD		SKCM		UVM	
	markers	Cor	*P*	Cor	*P*	Cor	*P*	Cor	*P*	Cor	*P*	Cor	*P*	Cor	*P*	Cor	*P*
NK cell	XCL1	0.574	**	0.292	**	0.2	***	0.548	***	0.359	***	0.459	***	0.673	***	0.324	*
	XCL2	0.564	**	0.266	**	0.516	***	0.559	***	0.395	***	0.606	***	0.71	***	0.4	**
	NCR1	0.577	**	0.155	0.0552	0.375	***	0.284	***	0.102	0.0204	0.421	***	0.482	***	0.113	0.32
Treg cell	FOXP3	0.85	***	0.136	0.0933	0.448	***	0.561	***	-0.011	0.802	0.612	***	0.558	***	0.39	**
Macrophage	CD68	0.554	**	0.23	*	0.124	*	0.208	***	0.425	***	0.453	***	0.292	***	-0.017	0.879
	CD84	0.763	***	0.075	0.357	0.395	***	0.285	***	0.292	***	0.543	***	0.603	***	0.398	**
	CD163	0.65	***	0.268	**	0.354	***	0.164	**	0.358	***	0.421	***	0.509	***	0.247	0.0272
	MS4A4A	0.774	***	0.271	**	0.447	***	0.265	***	0.326	***	0.496	***	0.558	***	0.238	0.0337
Dendritic cell	CCL13	0.63	***	0.303	**	0.285	***	0.113	*	0.039	0.373	0.393	***	0.348	***	-0.039	0.731
	CD209	0.745	***	-0.043	0.599	0.265	***	0.125	*	0.072	0.1	0.349	***	0.419	***	0.234	0.0368
	HSD11B1	0.238	0.162	0.344	***	0.33	***	0.172	***	-0.029	0.512	0.562	***	0.574	***	0.189	0.0927
Mast cell	TPSB2	0.592	**	0.14	0.0845	0.154	**	0	0.998	-0.077	0.0813	0.275	***	0.097	0.0346	-0.089	0.43
	TPSAB1	0.613	***	0.176	0.03	0.148	**	-0.023	0.599	-0.005	0.917	0.294	***	0.114	0.0133	0.046	0.683
	CPA3	0.562	**	0.147	0.0706	0.111	0.011	-0.084	0.0528	0.104	0.0178	0.259	***	0.161	**	0.037	0.746
	MS4A2	0.438	*	0.089	0.275	0.157	**	0.018	0.68	0.001	0.974	0.271	***	0.181	***	0.021	0.856
	HDC	0.566	**	0.112	0.17	0.265	***	0.241	***	0.231	***	0.403	***	0.261	***	0.116	0.306
Th1 cell	TBX21	0.826	***	-0.144	0.0757	0.667	***	0.455	***	0.373	***	0.691	***	0.721	***	0.363	**
Th2 cell	GATA3	0.782	***	0.081	0.317	0.307	***	0.322	***	0.436	***	0.473	***	0.595	***	0.346	*
	STAT6	0.072	0.675	0.105	0.196	0.003	0.946	0.178	***	0.313	***	0.027	0.542	-0.085	0.0665	-0.038	0.738
	STAT5A	0.476	*	0.157	0.053	0.478	***	0.413	***	0.418	***	0.573	***	0.108	0.0191	-0.517	***
	IL13	0.099	0.567	-0.034	0.672	0.368	***	0.395	***	0.094	0.0328	0.292	***	0.287	***	0.21	0.0617
Th17 cell	IL17A	0.395	0.0171	-0.004	0.961	0.191	***	0.13	*	0.07	0.11	0.315	***	-0.023	0.618	NA	NA
Tfh cell	CXCR5	0.469	*	0.088	0.28	0.511	***	0.412	***	0.355	***	0.587	***	0.559	***	0.291	*
	CXCL13	0.719	***	0.166	0.0406	0.586	***	0.636	***	-0.068	0.125	0.657	***	0.744	***	0.425	***
PMN-MDSC	ITGAM	0.379	0.0233	0.226	*	0.375	***	0.244	***	0.298	***	0.483	***	0.513	***	0.056	0.62
	CD33	0.735	***	0.294	**	0.468	***	0.456	***	0.373	***	0.576	***	0.56	***	0.238	0.0336
M-MDSC	CD14	0.54	**	0.34	***	0.37	***	0.265	***	0.34	***	0.501	***	0.525	***	0.179	0.112

NK cell, natural killer cell; Treg cell, regulatory T cell; Th, T helper cell; Tfh, Follicular helper T cell; PMN-MDSC, polymorphonuclear myeloid-derived suppressor cells; M-MDSC, monocytic myeloid-derived suppressor cells.

* *P*<0.01; ** *P*<0.001; *** *P*<0.0001.

TABLE S4 Correlation analysis between *MIR155HG* and immunoinhibitors and immunostimulators relate genes

	Gene markers	CHOL		GBM		HNSC		KIRC		LGG		LUAD		SKCM		UVM	
		Cor	P	Cor	P	Cor	P	Cor	P	Cor	P	Cor	P	Cor	P	Cor	P
	CD160	-0.042	0.81	-0.077	0.35	0.478	***	0.427	***	0.026	0.55	0.47	***	0.505	***	0.599	***
	CD96	0.837	***	0.2	0.013	0.602	***	0.636	***	0.471	***	0.692	***	0.707	***	0.416	**
	CSF1R	0.54	**	0.088	0.28	0.404	***	0.25	***	0.179	***	0.489	***	0.563	***	0.078	0.49
	CTLA4	0.772	***	0.11	0.175	0.656	***	0.743	***	0.357	***	0.788	***	0.533	***	0.405	**
	HAVCR2	0.74	***	0.252	*	0.558	***	0.118	*	0.412	***	0.639	***	0.678	***	0.388	**
	IDO1	0.842	***	0.305	**	0.556	***	0.251	***	0.442	***	0.52	***	0.676	***	0.506	***
	IL10	0.627	***	0.327	***	0.372	***	0.356	***	0.35	***	0.534	***	0.574	***	0.304	*
	IL10RB	0.164	0.34	0.397	***	0.158	**	0.277	***	0.473	***	0.244	***	0.322	***	0.453	***
	KIR2DL1	0.143	0.41	0.01	0.9	0.323	***	0.097	0.025	0.088	0.046	0.218	***	0.366	***	0.15	0.18
Immunoinhibitors	KIR2DL3	0.216	0.21	-0.009	0.91	0.394	***	0.143	**	0.136	*	0.308	***	0.47	***	0.195	0.08
	LAG3	0.66	***	0.117	0.149	0.588	***	0.681	***	0.303	***	0.613	***	0.694	***	0.501	***
	LGALS9	0.311	0.066	0.113	0.16	0.55	***	0.433	***	0.436	***	0.4	***	0.553	***	0.26	0.02
	PDCD1	0.485	*	0.113	0.164	0.656	***	0.673	***	0.507	***	0.682	***	0.695	***	0.476	***
	TGFB1	0.265	0.12	0.274	**	-0.157	**	0.177	***	0.395	***	0.34	***	0.3	***	0.134	0.24
	TGFBR1	0.038	0.83	0.116	0.152	-0.156	**	-0.062	0.152	0.382	***	0.126	*	0.016	0.73	0.29	*
	TIGIT	0.898	***	0.05	0.537	0.604	***	0.669	***	0.105	0.018	0.743	***	0.747	***	0.432	***
	VTCN1	-0.027	0.874	-0.284	**	0.07	0.108	-0.139	*	-0.143	*	-0.016	0.724	-0.153	**	0.237	0.0339
	CD274	0.507	*	0.255	*	0.282	***	0.116	*	0.28	***	0.508	***	0.588	***	0.342	*
	PDCD1LG2	0.76	***	0.371	***	0.303	***	0.373	***	0.447	***	0.633	***	0.67	***	0.36	*
	BTNL2	0.316	0.06	0.003	0.9	0.106	0.016	0.154	**	0.045	0.306	0.225	***	0.238	***	0.094	0.407
	CD27	0.85	***	0.187	0.02	0.575	***	0.657	***	0.336	***	0.657	***	0.695	***	0.5	***
	CD28	0.772	***	0.088	0.28	0.498	***	0.477	***	0.354	***	0.652	***	0.597	***	0.314	*
	CD40	0.381	0.022	0.281	**	0.453	***	0.384	***	0.42	***	0.556	***	0.557	***	0.011	0.93
	CD40LG	0.737	***	0.163	0.04	0.498	***	0.426	***	0.373	***	0.599	***	0.577	***	0.28	0.012
	CD48	0.851	***	0.267	**	0.628	***	0.536	***	0.486	***	0.757	***	0.715	***	0.553	***
	CD70	0.888	***	0.276	**	0.398	***	0.44	***	0.337	***	0.592	***	0.497	***	0.32	*
	CXCL12	0.288	0.089	0.034	0.68	0.249	***	-0.015	0.73	0.115	*	0.367	***	0.498	***	0.164	0.15
	CXCR4	0.804	***	0.264	**	0.505	***	0.403	***	0.371	***	0.591	***	0.518	***	0.39	**
	ICOS	0.901	***	0.153	0.058	0.553	***	0.699	***	0.494	***	0.774	***	0.73	***	0.387	**
Immunostimulators	IL6	0.507	*	0.356	***	0.071	0.103	0.201	***	0.439	***	0.232	***	0.311	***	0.489	***
	IL6R	-0.088	0.61	0.123	0.13	-0.249	***	-0.041	0.35	0.238	***	0.009	0.83	-0.026	0.58	0.289	*
	LTA	0.796	***	-0.085	0.3	0.61	***	0.682	***	0.172	***	0.751	***	0.67	***	0.305	*
	MICA	-0.006	0.97	0.405	***	0.143	*	0.186	***	0.327	***	0.147	**	-0.092	0.047	-0.058	0.61
	MICB	0.544	**	0.13	0.11	0.328	***	0.507	***	0.387	***	0.43	***	0.312	***	0.241	0.031
	RAET1E	0.131	0.445	0.026	0.75	-0.102	0.02	-0.099	0.02	0.152	**	0.11	0.012	0.134	*	0.234	0.037
	TMIGD2	0.521	*	0.091	0.265	0.515	***	0.342	***	0.003	0.944	0.6	***	0.486	***	-0.067	0.558
	ULBP1	-0.137	0.425	0.083	0.309	-0.07	0.108	0.089	0.041	0.286	***	0.055	0.22	0.135	*	0.521	***
	CD80	0.756	***	0.141	0.0827	0.445	***	0.537	***	0.455	***	0.64	***	0.69	***	0.379	**
	CD86	0.871	***	0.217	*	0.47	***	0.418	***	0.372	***	0.635	***	0.699	***	0.389	**

* $P<0.01$; ** $P<0.001$; *** $P<0.0001$.

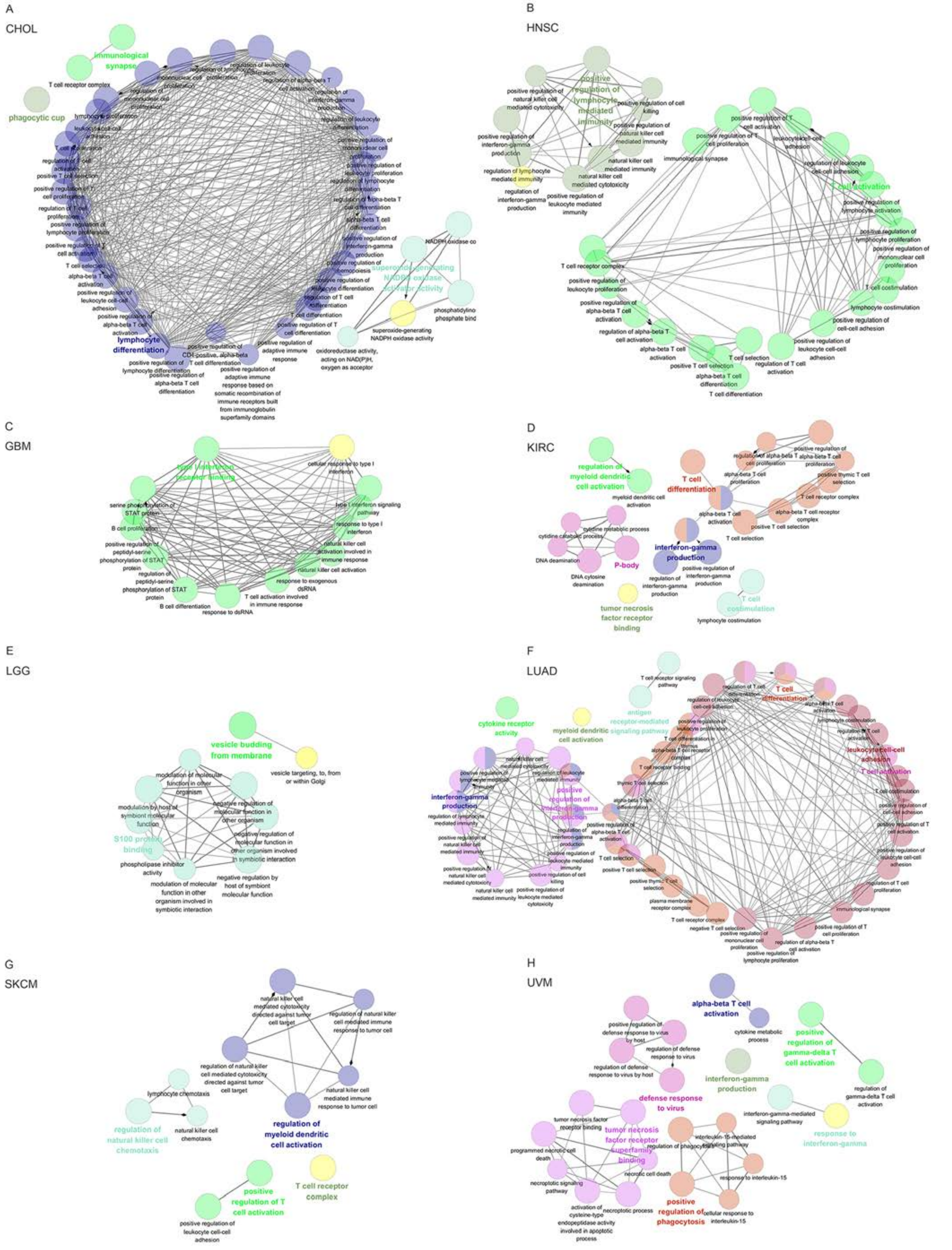

FIGURE S1 Gene Ontology (GO) analysis of the genes co-expression with *MIR155HG*. GO enrichment the mRNAs those were co-expressioned with *MIR155HG* in (**A**)CHOL, (**B**)HNSC, (**C**)GBM, (**D**)KIRC, (**E**)LGG, (**F**)LUAD, (**G**)SKCM, (**H**)UVM (show only pathways with pV≤0.001).

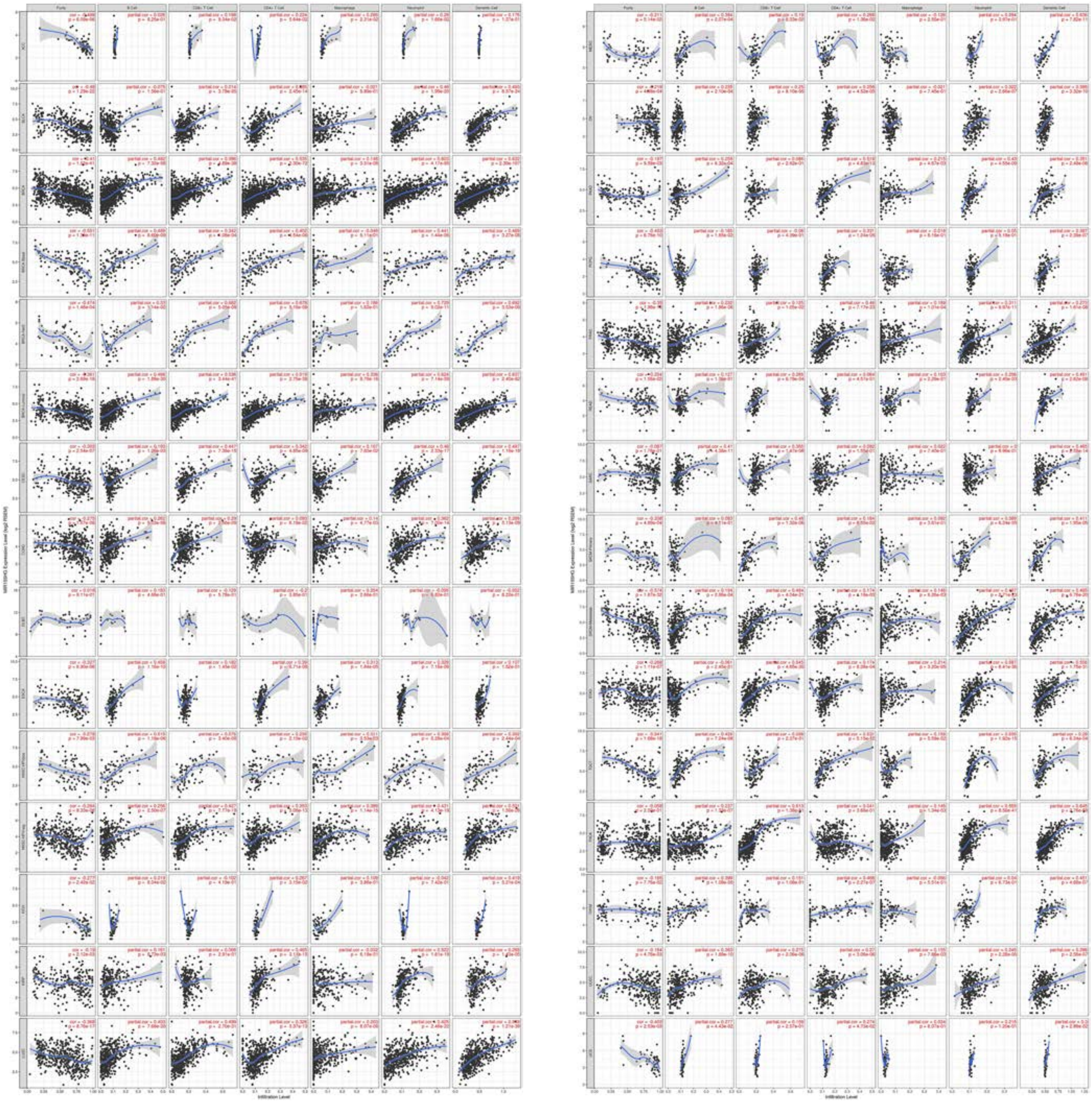

FIGURE S2 TIMER analysis of the immune cell co-expression with *MIR155HG* in cancers.

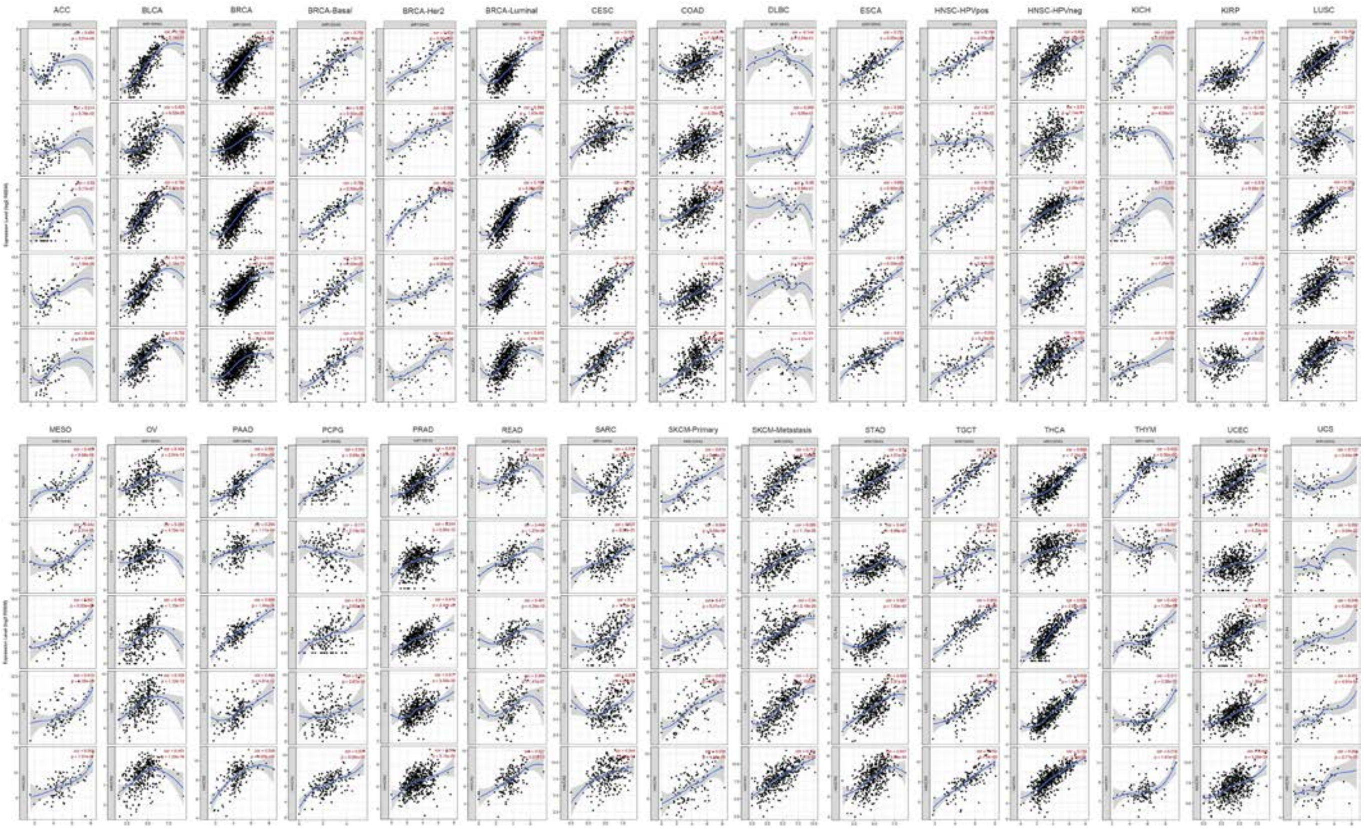

FIGURE S3 Correlation of *MIR155HG* expression with immune checkpoints molecules in cancers TIMER analysis.
